# An Early Role for Wnt Signaling in Specifying Neural Patterns of *Cdx* and *Hox* Gene Expression and Motor Neuron Subtype Identity

**DOI:** 10.1371/journal.pbio.0040252

**Published:** 2006-07-18

**Authors:** Ulrika Nordström, Esther Maier, Thomas M Jessell, Thomas Edlund

**Affiliations:** **1**Umeå Center for Molecular Medicine, Umeå University, Umeå, Sweden; **2**Howard Hughes Medical Institute and Center for Neurobiology and Behavior, Deparment of Biochemistry and Molecular Biophysics, Columbia University, New York, New York, United States of America; Stanford UniversityUnited States of America

## Abstract

The link between extrinsic signaling, progenitor cell specification and neuronal subtype identity is central to the developmental organization of the vertebrate central nervous system. In the hindbrain and spinal cord, distinctions in the rostrocaudal identity of progenitor cells are associated with the generation of different motor neuron subtypes. Two fundamental classes of motor neurons, those with dorsal (dMN) and ventral (vMN) exit points, are generated over largely non-overlapping rostrocaudal domains of the caudal neural tube. *Cdx* and *Hox* genes are important determinants of the rostrocaudal identity of neural progenitor cells, but the link between early patterning signals, neural *Cdx* and *Hox* gene expression, and the generation of dMN and vMN subtypes, is unclear. Using an in vitro assay of neural differentiation, we provide evidence that an early Wnt-based program is required to interact with a later retinoic acid- and fibroblast growth factor–mediated mechanism to generate a pattern of *Cdx* and *Hox* profiles characteristic of hindbrain and spinal cord progenitor cells that prefigure the generation of vMNs and dMNs.

## Abstract 2

Early Wnt signaling acts in combination with retinoic acid and/or FGF signals to induce the gene expression profiles forecasting differentiation of motor neurons characteristic of the hindbrain and spinal cord.

## Introduction

During the early development of the vertebrate central nervous system, the position of generation of post-mitotic neurons depends on the patterning of progenitor cells along the dorsoventral and rostrocaudal axes of the neural tube [ 1– 3]. At many levels of the neuraxis, the dorsoventral pattern of progenitor cells, which later gives rise to motor, sensory, and local circuit neurons, is initiated by the opponent signaling activities of Sonic hedgehog (Shh) and bone morphogenetic proteins [ 2, 4, 5]. In contrast, the rostrocaudal pattern of neural progenitor cells that differentiate into distinct neuronal subtypes is imposed, in part, by opponent retinoid and fibroblast growth factor (FGF) signals [ 6– 9]. Within the hindbrain and spinal cord, the rostrocaudal positional identity of neurons is reflected most clearly by the generation of different motor neuron (MN) subtypes. One fundamental distinction in MN subtype identity is the emergence of two major classes of MNs that exhibit distinctive axonal trajections, ventral exiting motor neurons (vMNs) and dorsal exiting motor neurons (dMNs) [ 10]. vMNs include most spinal MNs as well as hypoglossal and abducens MNs of the caudal hindbrain [ 1, 10, 11], whereas dMNs are found throughout the hindbrain and at cervical levels of the spinal cord [ 10]. Each of the many subsequent distinctions in MN subtype identity emerge through the diversification of these two basic neuronal classes [ 2].

Despite many advances in defining the mechanisms of MN diversification [ 7, 8, 12– 14], it remains unclear how neural progenitors in the hindbrain and spinal cord acquire a rostrocaudal positional character that results in the generation of dMN and vMN classes. At both hindbrain and spinal levels, *Hox* genes are informative markers of the rostrocaudal positional identity of progenitor cells. Within the hindbrain, distinct rhombomeres are delineated by the nested expression of 3′ *Hox* genes [ 3, 15], whereas the spinal expression of 5′ *Hox* genes distinguishes progenitor cells and post-mitotic neurons at cervical, brachial, thoracic, and lumbar levels [ 1, 6– 8, 13, 16]. Moreover, *Hox* genes are determinants of MN subtype identity in both hindbrain and spinal cord. In the hindbrain, for example, the restricted expression of Hoxb1 helps to determine the identity of facial MNs [ 1, 17– 20], and in the spinal cord the restricted expression of Hox6, Hox9, and Hox10 proteins establishes MN columnar subtype [ 7, 16]. In addition, a more complex Hox transcriptional regulatory network specifies spinal MN pool identity and connectivity [ 21]. The neural pattern of *Hox* expression is, in turn, regulated by members of the *Cdx* homeobox gene family [ 6, 22– 25]. *Cdx* genes are transiently expressed in the caudal-most region of the neural plate prior to the onset of 5′ *Hox* gene expression [ 26– 28] and appear to be direct regulators of the expression of 5′ *Hoxb* genes [ 6, 23, 24, 27]. Thus, analysis of spatial profiles of *Cdx* and *Hox* gene expression may provide clues about the identity of signals that pattern MN subtypes in the hindbrain and spinal cord.

Several recent studies have provided insight into the signals that impose rostrocaudally-restricted patterns of neural *Cdx* and *Hox* expression. Retinoic acid (RA) and FGF signals appear to have opponent roles in the rostrocaudal patterning of *Hox* gene expression in the caudal hindbrain (cHB) and spinal cord [ 6, 8]. Mesodermal-derived RA signals promote the expression of *Hox* genes characteristic of the cHB and rostral spinal cord (rSC) [ 11, 29, 30], whereas FGF signals pattern the expression of *Hox* genes at more caudal levels of the spinal cord. At an earlier developmental stage, neural progenitors have been shown to acquire caudal forebrain, midbrain, and rostral hindbrain positional identities in response to graded Wnt signaling at the gastrula stage [ 31, 32]. It is unclear, however, whether an early phase of Wnt signaling is also required to establish *Cdx* and *Hox* gene expression profiles characteristic of the cHB and spinal cord, in turn specifying the generation of dMN and vMN subtypes.

This study uses in vitro assays of neural cell differentiation to obtain evidence that early Wnt signaling does indeed have a crucial role in specifying the identity of hindbrain and spinal cord progenitor cells as revealed by profiles of *Cdx* and *Hox* gene expression. This early influence of Wnt signaling is later refined by retinoid and FGF signals to impart additional rostrocaudal distinctions in *Hox* expression that correlate tightly with the generation of dMNs and vMNs. Our findings therefore define a crucial early role for Wnt signaling in inducing profiles of *Cdx* and *Hox* expression that prefigure the differentiation of dMN and vMN subtypes in the developing hindbrain and spinal cord.

## Results

### Transcriptional Markers of Progenitor Cell Position and MN Subtype

To explore how progenitor cells of different rostrocaudal regional identity differentiate into dMNs and vMNs, we analyzed a panel of transcription factors that are expressed in different temporal and spatial patterns in the developing hindbrain and spinal cord.

**Figure 1:**
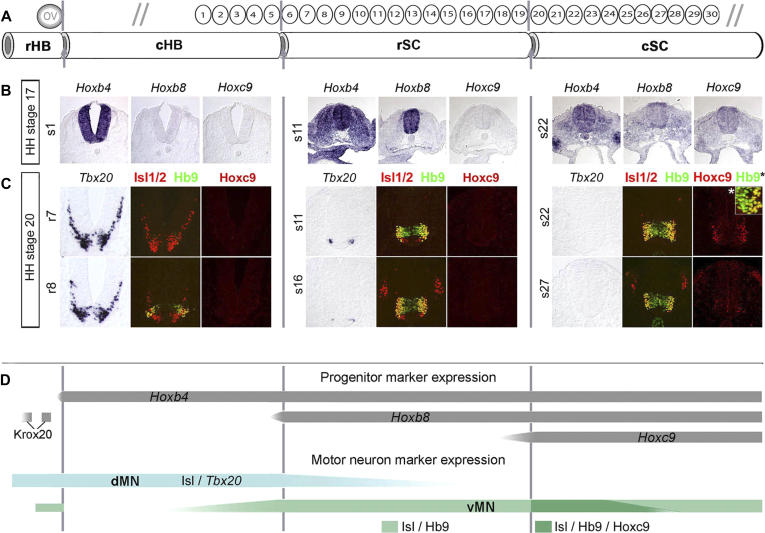
Profiles of *Hox* Gene Expression, dMNs, and vMNs in the Hindbrain and Spinal Cord (A) Schematic figure of the caudal embryonic neural tube divided into four distinct regions along the rostrocaudal axis: rHB; r7 and r8 defined as cHB; the region of the spinal cord located at the level of somite 6–19 defined as the rSC; and the region located caudal to somite 19 defined as cSC. (B) In a stage 17 (30-somite) chick embryo, the rostral borders of *Hoxb4, Hoxb8,* and *Hoxc9* expression are located just caudal to the otic vesicle (OV) (at the level of the r6/7 border), at the level of somite 5/6, and at the level of somite 19/20, respectively. *Hoxb4* expression in the absence of *Hoxb8* and *Hoxc9* is characteristic of cells in the cHB (r7 and r8). Expression of *Hoxb4* and *Hoxb8* in the absence of *Hoxc9* is characteristic of the rSC domain, and expression of *Hoxb4, Hoxb8, and Hoxc9* is characteristic of cells in the cSC domain. (C) In stage 20 (42-somite) chick embryos, the rostral boundaries of expression of *Hoxb4, Hoxb8,* and *Hoxc9* in the neural tube are maintained as in a stage 17 embryo (± 1-somite). *Tbx20 ^+^*/Isl ^+^ dMNs are present at high numbers in the cHB and at lower numbers in the rSC. No *Tbx20 ^+^*/Isl ^+^ dMNs are found in the cSC. In contrast, Hb9 ^+^/Isl ^+^ vMNs are present at high numbers in both rSC and cSC, and at lower numbers in r8 of the cHB. Hoxc9 protein is expressed in a subset of Hb9 ^+^/Isl ^+^ vMNs in the cSC and thus, distinguishes vMNs in the cSC from vMNs in the rSC. (D) Horizontal bars represent rostrocaudal restrictions (applied to Figure 1A) of marker genes expressed by neural progenitor cells in the stage 17 neural tube, and by MNs in stage 20 embryos.

In the hindbrain, progenitor cells do not express *Cdx* genes [ 22, 26]. Cells in rhombomeres (r)3 and r5—here defined as the rostral hindbrain (rHB)—express Krox20 (Figure1D, [ 33]) and generate both dMNs defined by the expression of *Tbx20 ^+^*/Isl ^+^ and vMNs defined by the expression of Hb9 ^+^/Isl ^+^ ( Figure 1D, [ 34– 36]). In contrast, progenitor cells in r7 and r8—here defined as the cHB—express *Hoxb4* but not *Hoxb8* or *Hoxc9* (termed ***Hoxb4 ^+^*** */b8* ^−^ */c9 ^−^* cells and all monitored by in situ hybridization) ( Figure 1A, 1B, and 1D) and generate *Tbx20 ^+^*/Isl ^+^ dMNs. ( Figure 1C, and 1D, [ 34– 36]).

**Figure 2:**
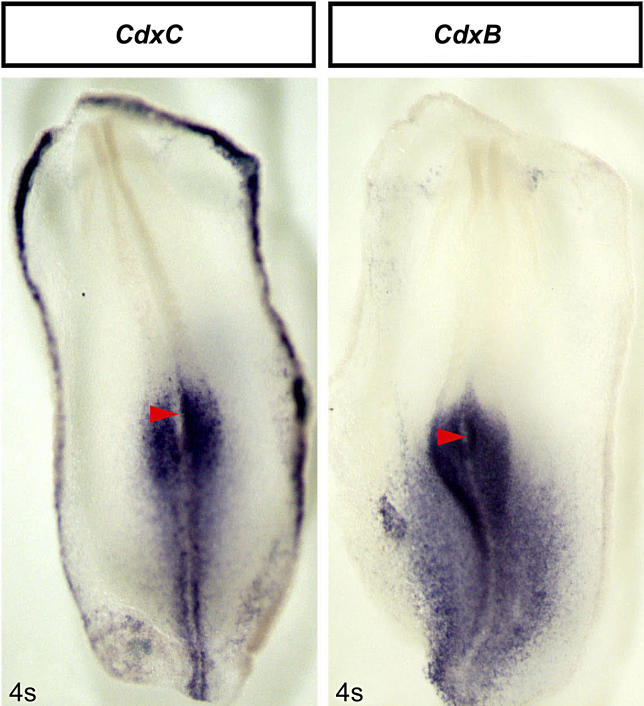
Expression Pattern of *CdxB* and *CdxC* Whole mount in situ hybridization of *CdxC* and *CdxB* in HH stage 8 embryos (4-somite). Expression of *CdxC* and *CdxB* is limited to levels adjacent and caudal to the node. The node is indicated by the red arrowhead.

Cells that give rise to the spinal cord can, at Hamburger and Hamilton (HH) stages 6–8, be defined by their profile of *CdxB* and *CdxC* expression ( Figure 2 and [ 22, 26]). Later, at stage 17, a complex spatial pattern of *Hox* gene expression defines progenitor cells of different regional identities ( Figure 1). Spinal progenitor cells at prospective cervical/brachial levels adjacent to somites 6–19 (here termed “rostral” spinal cord [rSC])—express *Hoxb4* and *Hoxb8,* but do not express *Hoxc9* (termed ***Hoxb4 ^+^/b8 ^+^/*** *c9 ^−^* cells) ( Figure 1A, 1B, and 1D). MN progenitor cells at this level generate Hb9 ^+^/Isl1 ^+^ vMNs, but only a few *Tbx20 ^+^*/Isl ^+^ dMNs ( Figure 1C, 1D and [ 34– 36]). Progenitor cells at thoracic levels of the spinal cord—adjacent to somite 20–30 (here termed “caudal” spinal cord [cSC])—express *Hoxb4, Hoxb8,* and *Hoxc9* (termed ***Hoxb4 ^+^/b8 ^+^/c9 ^+^*** cells) ( Figure 1A, 1B, and 1D) and generate Hb9 ^+^/Isl ^+^ vMNs that can be distinguished from those found at more rostral levels by their expression of Hoxc9 (Hb9 ^+^/Isl ^+^/Hoxc9 ^+^ MNs) ( Figure 1C and 1D, [ 7]). Since most of these markers are also expressed by non-neural cells, we used Sox proteins as general neural markers: Sox1 at stage 17 [ 37, 38] and Sox2 in combination with Sox3 at stage 8 as presumptive neural markers [ 39, 40]. In addition, we used Otx2 as a marker for neural cells located rostral to the midbrain/hindbrain boundary [ 41, 42].

### Hindbrain and Spinal Cord Progenitor Cells Acquire Rostrocaudal Regional Identity at Early Somite Stages

To study the patterning of *Cdx* and *Hox* genes by neural progenitor cells and its link to the differentiation of MN subtypes, we used in vitro differentiation assays that employed stage 4–8 prospective hindbrain and spinal cord explants, and stage 4 prospective forebrain (FB) explants. In stage 4 caudal (C) explants, cells have been exposed to caudalizing signals at the time of their isolation [ 31, 32], and these explants were used to examine the signals that specify hindbrain and spinal cord character. Cells in stage 4 FB explants have not been exposed to caudalizing signals at the time of their isolation [ 31, 32], and these explants were used in attempts to reconstitute more completely the events that direct the generation of dMNs and vMNs from “naive” neural cells.

**Figure 3:**
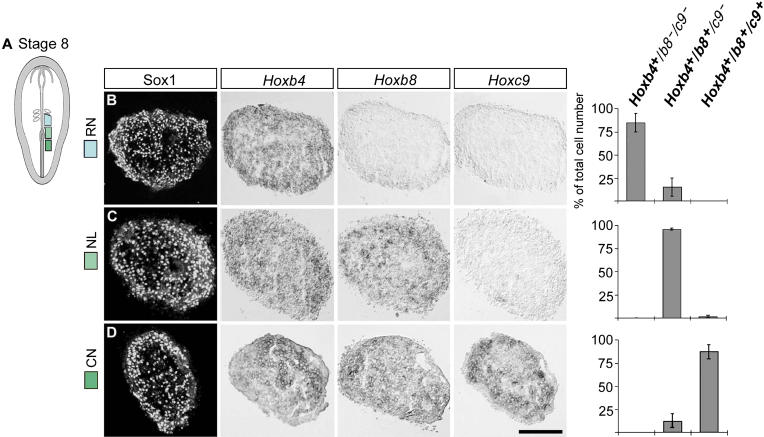
Hindbrain and Spinal Cord Progenitor Cells Acquire Rostrocaudal Regional Identity at the Early Somite Stage (A) Schematic drawing of a stage 8 chick embryo. The boxed regions indicate neural plate explants cultured in vitro for 40 h. (B–D) Sox1 was used as a general neural marker. Bars represent mean ± s.e.m. number of cells in ***Hoxb4 ^+^/*** *b8 ^−^/c9 ^−^,* ***Hoxb4 ^+^/b8 ^+^/*** *c9 ^−^,* and ***Hoxb4 ^+^/b8 ^+^/c9 ^+^*** domains, respectively, as percentage of total cell number. Each row represents consecutive sections from a single explant. (B) Explants isolated from the RN generated ***Hoxb4 ^+^/*** *b8 ^−^/c9 ^−^* cells and few ***Hoxb4 ^+^/b8 ^+^/ **c9 ^−^* cells ( *n* = 8 explants). (C) Explants isolated at the NL generated ***Hoxb4 ^+^/b8 ^+^/*** *c9 ^−^* cells ( *n* = 15 explants). (D) Explants isolated from the CN generated ***Hoxb4 ^+^/b8 ^+^/c9 ^+^*** and only a few ***Hoxb4 ^+^/b8 ^+^/*** *c9 ^−^* cells ( *n* = 12 explants). Scale bar represents 100 μm.

Prior to stage 8, the caudal neural plate is either specified as rSC or cSC (HH 5–7), but no explants generating cells of cHB character can be isolated (unpublished data). By stage 8, however, cells in explants isolated at a position just rostral to the regressing Hensen's node (RN explants) did not express *CdxB* and *CdxC* ( Figure 2) and generated cells characteristic of the cHB that expressed *Hoxb4* alone ( ***Hoxb4 ^+^/*** *b8 ^−^/c9 ^−^* cells (85 ± 10% of total cell number) after 40 h in culture ( Figure 3B). In contrast, cells in stage 8 explants isolated at the level of the node (NL explants) expressed *CdxB* and *CdxC* ( Figure 2). These explants also generated cells that expressed *Hoxb4* and *Hoxb8* in the absence of *Hoxc9* ( ***Hoxb4 ^+^/b8 ^+^/*** *c9 ^−^* cells; 96 ± 2% of total cell number), a marker profile characteristic of the rSC ( Figure 3C). Cells in stage 8 explants isolated caudal to the node (CN explants) also expressed *CdxB* and *CdxC* ( Figure 2), and generated ***Hoxb4 ^+^*/ *b8 ^+^*/ *c9 ^+^***cells (88 ± 7% of total cell number), a profile characteristic of the cSC ( Figure 3D). Thus by stage 8, prospective hindbrain and spinal cord progenitor cells appear to have acquired a coarse rostrocaudal regional identity.

**Figure 4:**
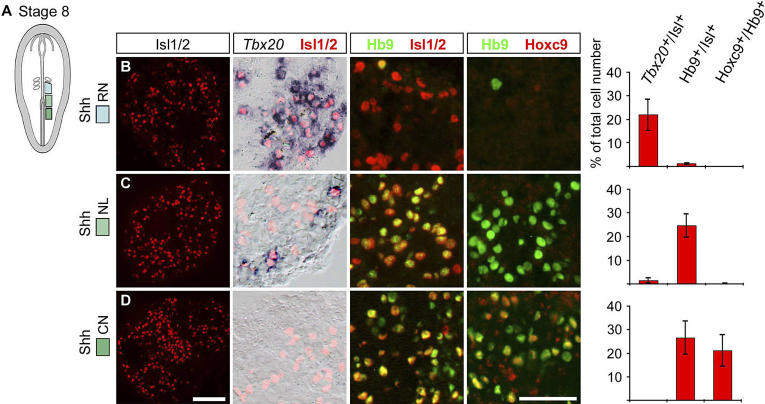
Hindbrain and Spinal Cord Progenitor Cells Generate dMNs and vMNs as Predicted by Their Respective *Hox* Expression Profile When Exposed to Shh-N (A) Schematic of a stage 8 chick embryo. The boxed regions indicate isolated neural plate explants cultivated alone for 4 h and then exposed to Shh-N (15 nM) for an additional ˜50 h. (B–D) Bars represent mean ± s.e.m. number of *Tbx20 ^+^*/Isl ^+^, Hb9 ^+^/Isl ^+^, and Hb9 ^+^/Hoxc9 ^+^ cells, respectively, as percentage of total cell number. Each row represents consecutive sections from a single explant. (B) Explants isolated from the RN generated *Tbx20 ^+^*/Isl ^+^ cells and a few Hb9 ^+^/Isl ^+^ cells ( *n* = 9 explants). (C) Explants isolated at the NL generated Hb9 ^+^/Isl ^+^ cells, only a few *Tbx20 ^+^*/Isl ^+^ cells, and no Hb9 ^+^/Isl ^+/^Hoxc9 ^+^ cells ( *n* = 12 explants). (D) Explants isolated from the CN generated Hb9 ^+^/Isl ^+^ cells of which 80 ± 6% expressed Hoxc9 but no *Tbx20 ^+^*/Isl ^+^ cells appeared ( *n* = 10 explants). Scale bars represent 100 μm (Isl) and 50 μm (double labels), respectively.

To determine whether the *Hox* gene profiles generated in stage 8 neural plate explants were correlated with the emergence of dMNs and vMNs, we exposed explants to the diffusible N-terminal fragment of the Shh protein (Shh-N) that exhibits MN-inducing activity [ 43, 44]. In the presence of Shh-N (15 nM) for ˜50 h, stage 8 RN explants generated many *Tbx20 ^+^*/Isl ^+^ dMNs (22 ± 7% of total cell number) and very few Hb9 ^+^/Isl ^+^ vMNs (1 ± 0.5% of total cell or number), a profile of MNs characteristic of the cHB (r7 and rostral r8) ( Figure 4B). Under these conditions, NL explants generated very few *Tbx20 ^+^*/Isl ^+^ dMNs (1.5 ± 1% of total cell number) and many Hb9 ^+^/Isl ^+^ vMNs (25 ± 4% of total cell number). Few, if any, of the induced Hb9 ^+^/Isl ^+^ vMNs co-expressed Hoxc9 (0.3 ± 0.3% of total cell number), indicative of an rSC positional character ( Figure 4C). CN explants did not generate *Tbx20 ^+^*/Isl ^+^ dMNs but did generate Hb9 ^+^/Isl ^+^ vMNs (27 ± 7% of total cell number), most of which expressed Hoxc9 protein (80 ± 6% of Hb9 ^+^ cells)—a profile characteristic of the thoracic spinal cord ( Figure 4D). Thus, by stage 8, progenitor cells that occupy different rostrocaudal positions within the caudal neural plate, defined in part by their *Cdx* profiles, are specified as hindbrain and spinal cord cells of either rostral or caudal regional character, and have acquired sufficient positional information to differentiate into dMNs and vMNs in a position-appropriate manner.

### Spinal Cord Cells of Caudal Character Are Specified at the Late Gastrula Stage

**Figure 5:**
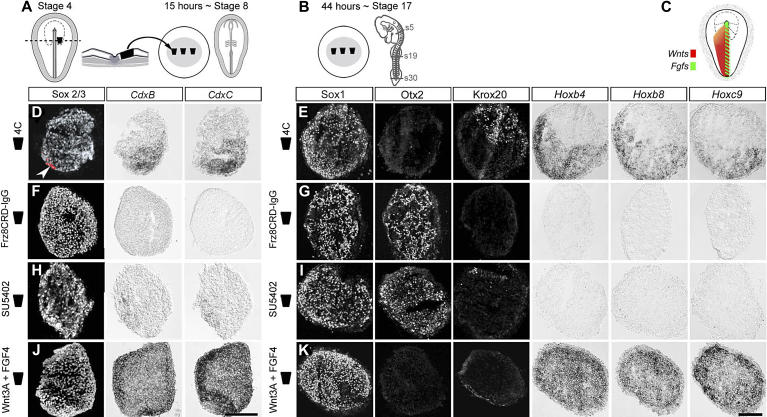
Cells of Spinal Cord Character Are Induced by Combinatorial Wnt and FGF Signaling at the Late Gastrula Stage (A and B) Caudal (C) neural plate tissue explants (black box) were isolated from HH stage 4 embryos and embedded in collagen matrix where their rostrocaudal orientation was maintained during in vitro cultivation for 15 h, corresponding to a stage 8 embryo (A, D, F, H, and J) or for 44 h, corresponding to a stage 17 ˜30-somite embryo (B, E, G, I, and K). (C) Schematic drawing indicating the expression pattern of *Wnt* (red) and *Fgf* genes (green) in the primitive streak and caudal ectoderm, and in the node and primitive streak, respectively. Black dotted line indicates the presumptive neural plate. (D–K) Each row represents consecutive sections from a single explant. (D, F, H, and J) Sox2/3 was used as a presumptive neural marker. (D) Stage 4 C explants were DiI-labeled and cultured alone. Cells in the caudal domain of the explants, close to the DiI-labeled cells, expressed *CdxB* and *CdxC* ( *n* = 12 explants). White arrowhead indicates the DiI-labeled cells. (E, G, I, and K) Sox1 was used as a general neural marker. (E) Cells in the rostral domain of 4 C explants cultured alone expressed Krox20, whereas ***Hoxb4 ^+^/b8 ^+^/c9 ^+^*** cells appeared in the caudal domain of the explants. A small domain of ***Hoxb4 ^+^/b8 ^+^/*** *c9 ^−^* cells, but no domain of cells expressing *Hoxb4* alone, were generated ( *n* = 30 explants). (F) Explants cultured in the presence of mFrz8CRD-IgG conditioned medium (300 μl/ml culture medium) generated Sox2/3 ^+^ neural cells but no *CdxB* or *CdxC* positive caudal neural cells ( *n* = 11 explants). (G) Explants cultured in the presence of mFrz8CRD-IgG conditioned medium (300 μl/ml culture medium) generated Otx2 ^+^ but no, or few, caudal neural cells ( *n* = 12 explants). (H) Explants cultured in the presence of SU5402 (5 μM), an inhibitor of FGF signaling, generated Sox2/3 ^+^ neural cells but no *CdxB* or *CdxC* positive caudal neural cells ( *n* = 8 explants). (I) Explants cultured in the presence of SU5402 (5 μM), an inhibitor of FGF signaling, generated Otx2 ^+^ but no, or few, caudal neural cells ( *n* = 9 explants). (J) Simultaneous exposure to Wnt3A (˜75 ng/ml) and FGF4 (60 ng/ml) resulted in the generation of cells that expressed *CdxB* and *CdxC* in the entire explant ( *n* = 17 explants). Scale bar represents 100 μm. (K) Exposure to Wnt3A (˜75 ng/ml) and FGF4 (60 ng/ml), in combination, almost completely blocked the generation of Krox20 ^+^ rHB cells, and only ***Hoxb4 ^+^/b8 ^+^/c9 ^+^*** spinal cord cells were generated ( *n* = 17 explants). Scale bar represents 100 μm.

To examine when the early pattern of *Cdx* and *Hox* profiles characteristic of hindbrain and spinal cord progenitor cells is established, we first monitored the generation of cells expressing *CdxB* and *CdxC* by in situ hybridization in stage 4 C explants cultured for 15 h in vitro, corresponding to a stage 8 embryo. We tracked the rostrocaudal orientation of these explants by labeling cells at the caudal margin with DiI crystals. Under these conditions, *CdxB ^+^* and *CdxC ^+^* cells (36 ± 11% of total cell number) were generated in a caudal domain of the explants ( Figure 5D), adjacent to the DiI labeling. To examine the rostrocaudal identity of the *Cdx ^+^* cells, we monitored the generation of *Hoxb4 ^+^, Hoxb8 ^+^,* and *Hoxc9 ^+^* cells by in situ hybridization in stage 4 C explants cultured for 44 h. Stage 4 C explants cultured for 44 h generated Krox20 ^+^ cells (45 ± 15% of total cell number), characteristic of the rHB away from the DiI label and, in a separate caudal domain (adjacent to the DiI label) ***Hoxb4 ^+^*/ *b8 ^+^*/ *c9 ^+^*** cells (52 ± 12% of total cell number) characteristic of the cSC ( Figures 7B and 5E). Thus, the generation of *CdxB ^+^/C ^+^* cells and ***Hoxb4 ^+^*/ *b8 ^+^*/ *c9 ^+^*** cells in a similar caudal domain of the explants supports the view that the expression of *Cdx* genes in neural plate cells is restricted to prospective spinal cord cells. In contrast, only a small domain of the explants generated ***Hoxb4 ^+^/b8 ^+^/*** *c9 ^−^* cells (12 ± 8% of total cell number), a marker profile characteristic of the rSC. Moreover, these explants lacked cells that expressed *Hoxb4* alone ( ***Hoxb4 ^+^/*** *b8 ^−^/c9 ^−^* cells) ( Figures 5E and 7B), characteristic of the cHB. These results provide evidence that, at stage 4, prospective caudal neural plate cells are specified as cells of rHB and cSC character, and only later acquire cHB or rSC character.

**Figure 6:**
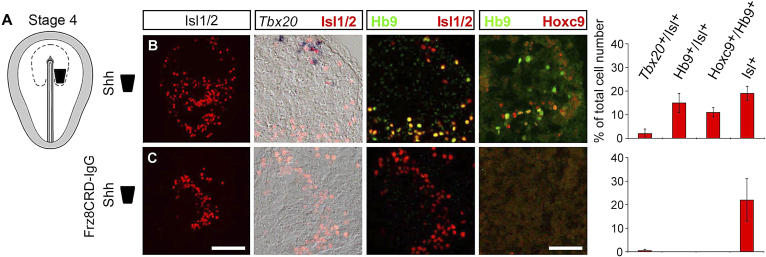
Wnt Signaling Is Required for the Generation of dMNs and vMNs in the Hindbrain and Spinal Cord (A) Caudal (C) neural plate tissue explants (black box) were isolated from HH stage 4 embryos. Explants were cultured alone or in the presence of mFrz8CRD-IgG for 28 h and then exposed to Shh-N (15 nM) for an additional 38 h. (B–C) Bars represent mean ± s.e.m. number of *Tbx20 ^+^*/Isl ^+^, Hb9 ^+^/Isl ^+^, Hb9 ^+^/Hoxc9 ^+^, and Isl1 ^+^ cells, respectively, as percentage of total cell number. Each row represents consecutive sections from a single explant. (B) Stage 4 C explants cultured with Shh-N alone generated *Tbx20 ^+^*/Isl ^+^ cells in the rostral domain of the explant and Hb9 ^+^/Isl ^+^ cells and Hb9 ^+^/Hoxc9 ^+^ cells in the caudal domain of the explant ( *n* = 18 explants). (C) Explants cultured in the presence of mFrz8CRD-IgG conditioned medium (500 μl/ml culture medium) and Shh-N generated Isl1/2 ^+^ cells but no *Tbx20 ^+^,* Hb9 ^+^, or Hoxc9 ^+^ cells ( *n* = 7 explants). Scale bars represent 100 μm (Isl1/2) and 50 μm (double labels), respectively.

We next examined whether prospective rHB and cSC cells have acquired sufficient rostrocaudal positional information by stage 4 to permit them to differentiate into dMNs or vMNs when exposed to Shh-N. To test this possibility, we cultured stage 4 C explants for 28 h to allow cells to acquire a stable rostrocaudal positional identity, and then for an additional 38 h in the presence of Shh-N (15 nM) (combined culture time corresponding to a stage 20, ˜40-somite embryo). In the presence of Shh-N, *Tbx20 ^+^*/Isl ^+^ dMNs (1 ± 1% of total cell number) and Hb9 ^+^/Isl ^+^ vMNs (16 ± 2% of total cell number) were generated in separate domains of the explants ( Figure 6B). A majority of the vMNs expressed Hoxc9 (10 ± 2% of total cell number)—a profile characteristic of vMNs at thoracic levels of the spinal cord ( Figure 6B). These results indicate that by stage 4, cells in the prospective caudal neural plate have acquired a positional character that permits them to differentiate into dMNs and vMNs.

**Figure 7:**
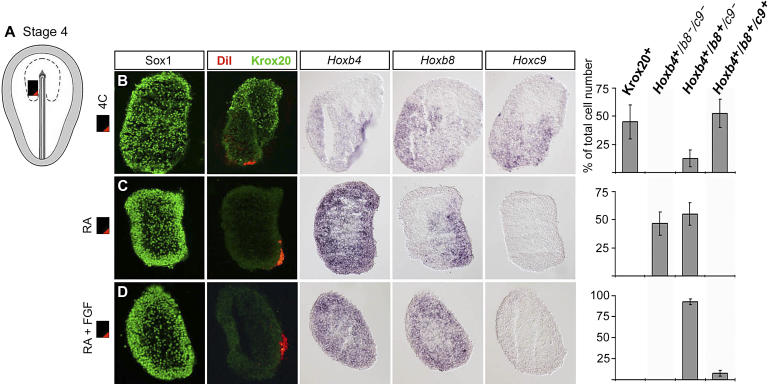
RA Induces cHB Cells, and RA and FGF, in Combination, Induce rSC Cells (A) Schematic drawing of a stage 4 embryo. Dotted line indicates the presumptive neural plate. Black box indicates caudal (C) neural plate explant isolated and cultured in vitro for 44 h. The red mark indicates the caudal margin of the explant labeled with DiI. Bars represent mean ± s.e.m. number of cells in Krox20 ^+^ ***Hoxb4 ^+^*** */b8 ^−^/c9 ^−^,* ***Hoxb4 ^+^/b8 ^+^/*** *c9 ^−^,* and ***Hoxb4 ^+^/b8 ^+^/c9 ^+^*** domains, respectively, as percentage of total cell number. (B–D) Each row represents consecutive sections from a single explant. (B) Control stage 4 C explants generated **Krox20 ^+^** cells in the rostral, and ***Hoxb4 ^+^/b8 ^+^*/ *c9 ^+^*** cells were generated in the caudal region of the explant, adjacent to the DiI-labeled cells. A small domain of ***Hoxb4 ^+^/b8 ^+^/*** *c9 ^−^* cells was generated in the medial region but no domain of cells expressing *Hoxb4* alone was generated ( *n* = 7 explants). (C) RA (10 nM) blocked the generation of Krox20 ^+^ and induced ***Hoxb4 ^+^/*** *b8 ^−^/c9 ^−^*cells in the rostral region. Adjacent to the DiI-labeled cells in the caudal region of the explant, ***Hoxb4 ^+^/b8 ^+^/*** *c9 ^−^* cells, but no ***Hoxb4 ^+^/b8 ^+^/c9 ^+^*** cells, were generated ( *n* = 6 explants). (D) RA (10 nM) and FGF4 (30 ng/ml), in combination, generated ***Hoxb4 ^+^/b8 ^+^*** */c9 ^−^* cells in both the rostral and caudal regions. No, or a few, ***Hoxb4 ^+^/b8 ^+^*/ *c9 ^+^*** cells appeared, and no Krox20 ^+^ or ***Hoxb4 ^+^*** */b8 ^−^/c9 ^−^* cells were generated ( *n* = 5 explants). Scale bar represents 100 μm.

### Joint Wnt and FGF Signaling at Late Gastrula Stages Specifies Spinal Cord Character

We next addressed the identity of the secreted signals that might be involved in the early specification of cells of spinal cord character. Several *Wnt* and *Fgf* genes are expressed in and around the prospective caudal neural plate [ 45– 47], and the specification of rHB cells at stage 4 requires convergent Wnt and FGF signaling [ 32, 48]. To examine whether combined Wnt and FGF signaling is required for the specification of cells of spinal cord positional character, we cultured stage 4 C explants in the presence of a soluble fragment of the Frizzled receptor 8 protein (Frz8CRD-IgG), an antagonist of Wnt signals [ 32, 49, 50], or with SU5402, an antagonist of FGF receptor signaling [ 49, 51, 52] and monitored the expression of *Cdx* and *Hox* genes.

In the presence of Frz8CRD-IgG or SU5402 (5 μM) for 15 h, the expression of both *CdxB* and *CdxC* was blocked ( Figure 5F and 5H). After 44 h under these conditions, the generation of cells of both rHB (Krox20 ^+^) and of spinal cord *(* ***Hoxb4 ^+^/b8 ^+^/*** *c9 ^−^* and ***Hoxb4 ^+^/b8 ^+^/c9 ^+^*** *)* character was almost completely blocked (3 ± 3% caudal cells remaining versus 64 ± 10% in the controls) ( Figure 5G and 5I). Instead, Otx2 ^+^ forebrain-like cells were generated (79 ± 9% of total cell number versus 0% in the controls) ( Figure 5G and 5I). These results support the idea that the specification of cells of spinal cord positional character also involves convergent Wnt and FGF signaling.

To test whether Wnt signaling in prospective rHB and cSC cells is required for the generation of vMNs and dMNs, stage 4 C explants were cultured in the presence of mFrz8CRD-IgG, and after 28 h Shh-N (15 nM) was added for a further 38 h. Under these conditions, the generation of *Tbx20* ^+^/Isl ^+^ dMNs and Hb9 ^+^/Isl ^+^ and Hoxc9 ^+^/Hb9 ^+^ vMNs was blocked (0% of total cell number), and instead **Isl ^+^**/ *Tbx20* ^−^/Hb9 ^−^/Hoxc9 ^−^ neurons, characteristic of the ventral forebrain [ 53, 54], were generated (18 ± 3% of total cell number) ( Figure 6C). Thus, exposure of prospective caudal neural plate cells to Wnt signals is required for the generation of vMNs and dMNs.

### High-Level Wnt Signaling Promotes Spinal Cord Character

We next examined whether differences in the level or duration of exposure to Wnt and FGF signals contribute to the early distinction in hindbrain and spinal cord character. To test this possibility, we exposed stage 4 C explants to exogenous Wnt and FGF signals for 15 h or 44 h in vitro. In explants exposed to Wnt3A (75 ng/ml) and FGF4 (60 ng/ml) simultaneously for 15 h, *CdxB* ^+^ and *CdxC ^+^* cells were generated throughout the entire explant ( Figure 5J). After 44 h of culture under these conditions, the generation of Krox20 ^+^ cells was largely suppressed (3 ± 2% of total cell number versus 45 ± 15% in the controls) and most cells acquired a ***Hoxb4 ^+^/b8 ^+^/c9 ^+^*** cSC character (96 ± 2% of total cell number versus 52 ± 12% in the controls) ( Figure 5K). To examine whether mesodermal cells were generated under these conditions, we monitored the expression of *Mox1*[ 55], which is expressed in caudal paraxial mesoderm and of *Brachyury (Bra)* [ 56], which at caudal levels is expressed in both the mesoderm and in cells of the forming caudal neural plate ( Figure S3G). No *Mox1* cells and no, or very few, *Bra ^+^* cells were induced ( Figure S3D), indicating that the few *Bra ^+^* cells represent caudal neural cells and not mesodermal cells. Exposure to FGF4 (60–120 ng/ml), or Wnt3A (150 ng/ml) alone did not increase the number of cells expressing *Cdx* genes, nor did it change the ratio of Krox20 ^+^ and ***Hoxb4 ^+^/b8 ^+^/c9 ^+^*** cells ( Figure S1 and unpublished data). These results are consistent with the view that exposure of cells to prolonged or higher level Wnt and FGF signaling promotes the specification of cells of spinal cord rather than midbrain or hindbrain character.

### RA Imparts Caudal Character to Hindbrain Cells and Rostral Character to Spinal Cord Cells

How then, are rostral and caudal sub-domains of the hindbrain and spinal cord established? Since Wnt signaling contributes to the distinction in specification of prospective rHB and cSC cells at gastrulation stages, we examined first whether exposure of prospective hindbrain and spinal cord cells to Wnt signals beyond stage 8 was required for the later specification of cHB and rSC cells. Stage 8 caudal neural plate explants exposed to Frz8CRD-IgG still generated cells of cHB and spinal cord character (unpublished data); indicating that prolonged exposure to Wnt signals is not required for the generation of these two sub-domains of the caudal neural tube.

By stage 8, the retinoid acid synthesizing enzyme retinaldehyde dehydrogenase 2 (RALDH2) is expressed in the paraxial mesoderm adjacent to the prospective cHB and rSC [ 57– 59]. RA signaling might therefore promote the generation of cells of cHB and rSC character by acting on neural cells that have already acquired an initial caudal character, through convergent Wnt and FGF signaling. To test this idea, we exposed stage 4 C explants to RA, and used the combinatorial expression of *Hoxb4, Hoxb8,* and *Hoxc9* to distinguish cells of cHB, and rSC or cSC character. Since *Cdx* expression does not distinguish between cells of rSC and cSC character, we did not monitor *Cdx* expression in these experiments. To map prospective rHB and cSC cells in these explants, we tracked their rostrocaudal orientation by labeling cells at the caudal margin with DiI crystals. We found that ***Hoxb4 ^+^*/ *b8 ^+^*/ *c9 ^+^*** cells derive from the caudal-most region of the explants, whereas Krox20 ^+^ cells derive from a rostral domain of the explants ( Figure 7B).

In the presence of RA (10 nM), no Krox20 ^+^ cells of rHB character were generated in the rostral domain of the explants—the domain lacking DiI-labeled cells. Instead, ***Hoxb4 ^+^/*** *b8 ^−^/c9 ^−^* cells characteristic of the cHB (47 ± 10% of total cell number versus 0% in the controls) were generated in this domain ( Figure 7C). In the caudal domain of the explants—the domain adjacent to DiI-labeled cells—the presence of RA (10 nM) blocked the generation of ***Hoxb4 ^+^/b8 ^+^/c9 ^+^*** cells characteristic of the cSC (0% of total cell number versus 52 ± 12% in controls), and instead, ***Hoxb4 ^+^/b8 ^+^/*** *c9 ^−^* cells characteristic of the rSC (55 ± 10% of total cell number versus 12 ± 8% in controls) were generated ( Figure 7C). Furthermore, RA was unable to induce caudal character in stage 4 prospective FB cells that had not been exposed to caudalizing signals [ 31] ( Figure S2E). These results provide evidence that RA promotes the generation of ***Hoxb4 ^+^/*** *b8 ^−^/c9 ^−^* cHB cells by blocking Krox20 and inducing *Hoxb4* expression in caudalized prospective Krox20 ^+^ rHB cells. In addition, they show that RA induces ***Hoxb4 ^+^/b8 ^+^/*** *c9 ^−^* rSC cells by preventing *Hoxc9* expression in prospective cSC cells.

### FGF Signaling Contributes to the Distinction between Cells of cHB and rSC Character

We next examined how the distinction between cHB and rSC cells is established. RA promotes the generation of cHB cells, and FGFs promote the expression of *Hox* genes characteristic of the caudal region of the spinal cord [ 8, 46]. Moreover, RA and FGF signals act in an opponent manner during rostrocaudal patterning of *Hox* gene expression and MN progenitor cell specification [ 6, 8]. These observations led us to examine the possibility that FGF and RA signals converge during the initial assignment of cHB and rSC positional character.

We exposed stage 4 C explants to both RA (10 nM) and FGF4 (30 ng/ml) and assayed their *Hox* profile after 44 h. The generation of ***Hoxb4 ^+^/*** *b8 ^−^/c9 ^−^* cHB cells was suppressed (1 ± 1% of total cell number versus 47 ± 10% in explants cultured with RA alone), and most cells (92 ± 3% of total cell number versus 55 ± 10% in explants cultured with RA alone) expressed *Hoxb4* and *Hoxb8,* a profile indicative of rSC character. A few ***Hoxb4 ^+^*/ *b8 ^+^*/ *c9 ^+^*** cells (7 ± 4% of total cell number versus 0% in explants cultured with RA alone), characteristic of cSC character were also detected ( Figure 7D). Thus, in the presence of RA, prolonged exposure to FGF signals promotes the generation of cells of rSC at the expense of cHB character. This finding supports the idea that the status of FGF signaling biases whether RA exposure induces *Hox* gene profiles characteristic of cHB or rSC.

### Combinatorial Wnt, RA, and FGF Signals Impose Hindbrain and Spinal Cord Character in Naive Neural Cells

As a further test of the sufficiency of Wnt, FGF, and RA signals in establishing hindbrain and spinal cord pattern, we examined whether a combination of these factors can establish appropriate *Cdx* and *Hox* gene profiles in naive rostral forebrain cells that appear not to have been exposed to caudalizing signals in ovo.

**Figure 8:**
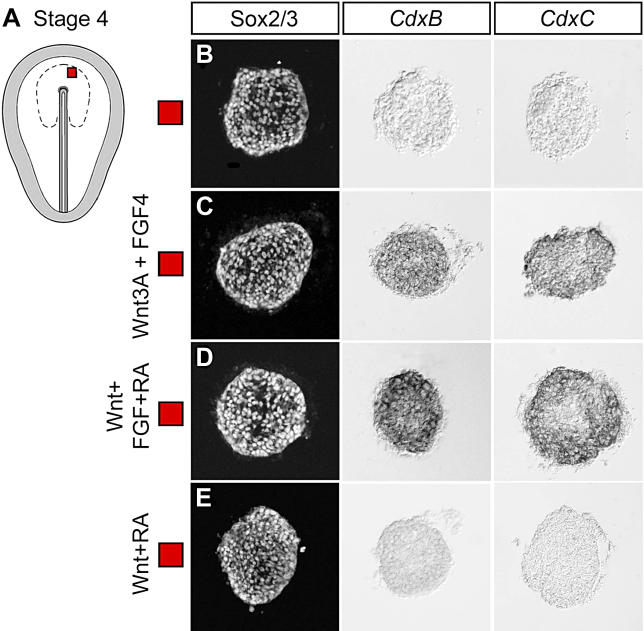
Wnt and FGF, in Combination, Induce *Cdx* Gene Expression in Prospective FB Cells (A) Schematic drawing of a stage 4 embryo. Dotted line indicates the presumptive neural plate. Red box indicates prospective FB explants used for in vitro studies. (B–D) Sox2 and Sox3, in combination, were used as general presumptive neural markers. Each row represents consecutive sections from a single explant. (B) Control stage 4 FB explants generated Sox2 ^+^/3 ^+^ but no *CdxC ^+^/CdxB* ^+^ presumptive neural cells ( *n* = 24 explants). (C) Stage 4 FB explants cultured in the presence of Wnt (˜150 ng/ml) and FGF (60 ng/ml) simultaneously generated *CdxC ^+^* */CdxB ^+^* presumptive caudal neural cells ( *n* = 26 explants). (D) 4 FB explants cultivated in the presence of Wnt3A (˜150 ng/ml), RA (10 nM), and FGF (30 ng/ml) generated *CdxC ^+^* */CdxB ^+^* presumptive caudal neural cells ( *n* = 18 explants). (E) 4 FB explants cultivated in the presence of Wnt3A (˜150 ng/ml) and RA (10 nM) did not generate *CdxC ^+^* */CdxB ^+^* presumptive caudal neural cells ( *n* =14 explants).

**Figure 9:**
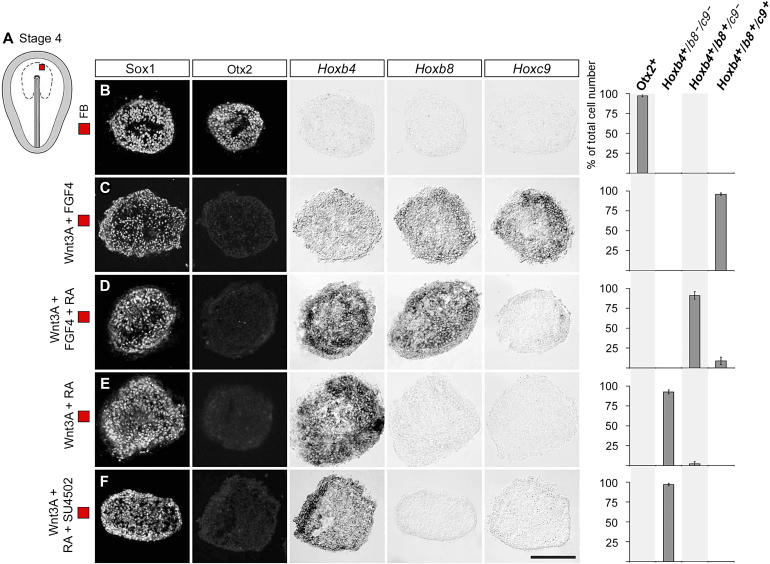
Combinatorial Wnt, RA, and FGF Signaling Reconstruct *Hox* Gene Profiles Characteristic of the cHB and Spinal Cord (A) Schematic drawing of a stage 4 chick embryo. Dotted line indicates the presumptive neural plate. Red box indicates FB explants isolated and cultured in vitro for 44 h. (B–F) Sox1 was used as a general neural marker. Bars represent mean ± s.e.m. number of cells in Otx2 ^+^, ***Hoxb4 ^+^/*** *b8 ^−^/c9 ^−^,* ***Hoxb4 ^+^/b8 ^+^/*** *c9 ^−^,* and ***Hoxb4 ^+^/b8 ^+^/c9 ^+^*** domains, respectively, as percentage of total cell number. Each row represents consecutive sections from a single explant. (B) Control stage 4 FB explants generated Sox1 ^+^/Otx2 ^+^ but no caudal neural cells ( *n* = 24 explants). (C) Stage 4 FB explants cultured in the presence of Wnt (˜150 ng/ml) and FGF (60 ng/ml) generated ***Hoxb4 ^+^/b8 ^+^/c9 ^+^*** cells and only a few Krox20 ^+^ cells ( *n* = 24 explants). (D) Cultivation in the presence of Wnt3A (˜150 ng/ml), RA (10 nM), and FGF (30 ng/ml) generated ***Hoxb4 ^+^/b8 ^+^/*** *c9 ^−^* cells and a few ***Hoxb4 ^+^/b8 ^+^/c9 ^+^*** cells ( *n* = 18 explants). (E) Cultivation in the presence of Wnt3A (˜150 ng/ml) and RA (10 nM) generated ***Hoxb4 ^+^/*** *b8 ^−^/c9 ^−^* cells and no, or only a few, ***Hoxb4 ^+^/b8 ^+^/*** *c9 ^−^* cells ( *n* = 28 explants). (F) Exposure to Wnt3A (˜150 ng/ml) and RA (10 nM) in the presence of SU5402 (3 μM), an inhibitor of FGF signaling, generated ***Hoxb4 ^+^/*** *b8 ^−^/c9 ^−^* cells ( *n* = 12 explants). Scale bar represents 100 μm.

Stage 4 FB explants cultured alone for 44 h generated Sox1 ^+^/Otx2 ^+^ cells (94 ± 4% of total cell number) ( Figures 9B and S2B). Wnt3A, FGF4, or RA added separately, or FGF4 and RA added in combination, did not induce cells of caudal neural character ( Figure S2; see also [ 31, 32]). Stage 4 FB explants exposed to Wnt3A and FGF4 in combination for 15 h, corresponding to stage 8, generated *CdxB ^+^* and *CdxC ^+^* cells (95 ± 4 % of total cell number) ( Figure 8C). After 44 h of culture, ***Hoxb4 ^+^/b8 ^+^/c9 ^+^*** cSC cells (96 ± 2% of total cell number) and only a few Krox20 ^+^ cells (4 ± 1% of total cell number) were generated ( Figure 9C). Thus, combined Wnt and FGF signals establish *Cdx* and *Hox* gene profiles indicative of cSC identity in prospective FB cells.

We next examined whether, in the presence of Wnt and FGF signals, RA can induce the generation of cHB or rSC cells. In the combined presence of Wnt3A (150 ng/ml), FGF4 (30 ng/ml), and RA (10 nM) for 15 h, stage 4 FB explants generated *CdxB ^+^* and *CdxC ^+^* cells (97 ± 3% of total cell number) ( Figure 8D). After 44 h of culture, ***Hoxb4 ^+^/b8 ^+^/*** *c9 ^−^* rSC cells (91 ± 5% of total cell number) and some ***Hoxb4 ^+^*/ *b8 ^+^/ *c9 *^+^*** cSC cells (9 ± 5% of total cell number), but no ***Hoxb4 ^+^/*** *b8 ^−^/c9 ^−^* cells of cHB character, were generated ( Figure 9D). Thus, Wnt signals in combination with RA and FGF signals predominantly induce a *Hox* gene profile characteristic of the rSC.

Next, we tested whether combined exposure to Wnt3A (150 ng/ml) and RA (10 nM) induces cHB cells. No *CdxB ^+^* and *CdxC ^+^* cells were generated after 15 h of culture ( Figure 8E). After 44 h of culture, many ***Hoxb4 ^+^/*** *b8 ^−^/c9 ^−^* cHB cells (92 ± 3% of total cell number versus 0% in control explants), few (3 ± 3% of total cell number) ***Hoxb4 ^+^/b8 ^+^/*** *c9 ^−^,* and no ***Hoxb4 ^+^*/ *b8 ^+^*/ *c9 ^+^*** cells characteristic of the rSC and cSC, were generated ( Figure 9E). Moreover, in the presence of SU5402 (3 μM), Wnt3A (150 ng/ml) and RA (10 nM) still induced ***Hoxb4 ^+^/*** *b8 ^−^/c9 ^−^/* cHB cells (97 ± 2% of total cell number) but no ***Hoxb4 ^+^/b8 ^+^/*** *c9 ^−^* cells, suggesting that the convergent activities of Wnt and RA signaling are sufficient to induce cHB cells ( Figure 9F). No combination of Wnt3A-, RA-, and FGF4-induced *Mox1 ^+^* cells characteristic of caudal paraxial mesoderm, and only Wnt3A and FGF4 in combination, induced a few *Bra ^+^* cells ( Figure S3B and S3C, unpublished data). These results provide evidence that Wnt signals, in combination with RA and/or FGF, are sufficient to reconstruct *Cdx* and *Hox* gene profiles indicative of cHB and both rSC and cSC in naive prospective FB explants.

**Figure 10:**
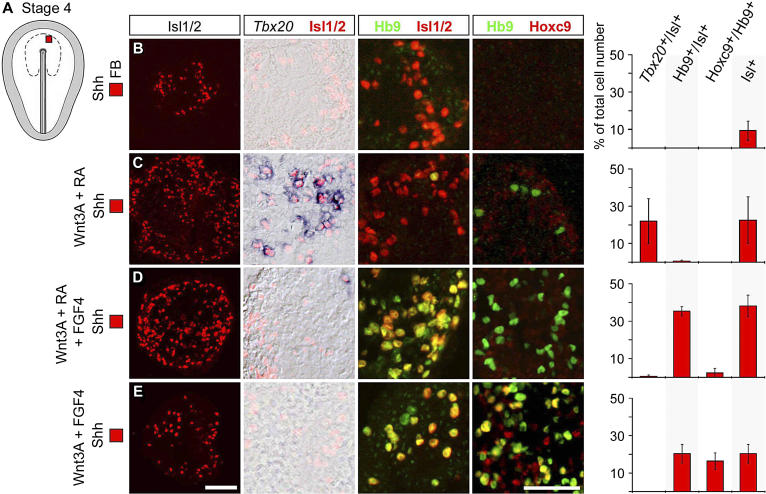
Hox Gene Profiles Induced by Wnt, RA, and/or FGF Signals Predict Later MN Subtype (A) Schematic drawing of a stage 4 embryo. Dotted line indicates the presumptive neural plate. Red box indicates prospective FB explants used for in vitro studies. (B–E) Explants were cultured alone or exposed to Wnt, RA, and/or FGF4 for 44 h, then washed and exposed to Shh-N (15 nM) for an additional 22 h. Bars represent mean ± s.e.m. number of *Tbx20 ^+^*/Isl ^+^, Hb9 ^+^/Isl ^+^, and Hb9 ^+^/Hoxc9 ^+^ cells, respectively, as percentage of total cell number. (B–E) Each row represents consecutive sections from a single explant. (B) Stage 4 FB explants cultured alone, before exposure of Shh-N, generated Isl ^+^ cells but no *Tbx20 ^+^,* Hb9 ^+^, or Hoxc9 ^+^ cells ( *n* = 6 explants). (C) Stage 4 FB explants cultured in the presence of Wnt3A (˜150 ng/ml) and RA (10 nM), before exposure of Shh-N, generated *Tbx20 ^+^*/Isl ^+^ cells but no, or very few, Hb9 ^+^/Isl ^+^ cells and no Hoxc9 ^+^ cells ( *n* = 12 explants). (D) Cultivation in the presence of Wnt3A (˜150 ng/ml), RA (10 nM), and FGF4 (30 ng/ml), before exposure of Shh-N, generated Hb9 ^+^/Isl ^+^ cells and only a few *Tbx20* ^+^/Isl ^+^ and Hb9 ^+^/Hoxc9 ^+^ cells ( *n* = 14 explants). (E) Cultivation in the presence of Wnt3A (˜150 ng/ml) and FGF4 (60 ng/ml), before exposure of Shh-N, generated Hb9 ^+^/Isl ^+^ and Hb9 ^+^/Hoxc9 ^+^ cells but no *Tbx20 ^+^* cells ( *n* = 9 explants). Scale bars represent 100 μm (Isl) and 50 μm (double labels), respectively.

We also addressed whether *Hox* gene profiles induced by Wnt, RA, and/or FGF signals in naive prospective FB cells predict the later generation of dMNs and vMNs. We cultivated stage 4 FB explants in the presence of Wnt, RA, and/or FGF signals for 44 h, to allow hindbrain and spinal cord progenitor cells to acquire their rostrocaudal positional identity, and for an additional 22 h in the absence or presence of Shh-N (15 nM) to induce MN differentiation. Stage 4 FB explants, grown both in the absence or presence of Shh-N (15 nM), generated Otx2 ^+^ neural progenitor cells and **Isl ^+^**/ *Tbx20* ^−^/Hb9 ^−^/Hoxc9 ^−^ ventral FB neurons ( Figure 10B and Table 1, [ 54]). Stage 4 FB cells exposed to FGF4 (60 ng/ml) and Wnt3A (150 ng/ml), which generate ***Hoxb4 ^+^/b8 ^+^/c9 ^+^*** cSC progenitor cells, generated Hb9 ^+^/Isl ^+^ and Hoxc9 ^+^/Hb9 ^+^ vMNs in the presence of Shh-N (15nM) ( Figure 10E and Table 1). We also determined which MN subtypes are generated from the ***Hoxb4 ^+^/b8 ^+^/*** *c9 ^−^* rSC progenitor cells, induced by Wnt3A (150 ng/ml), FGF4 (30 ng/ml), and RA (10 nM). Addition of Shh-N (15 nM) induced a large number of Hb9 ^+^/Isl ^+^ MNs, but few Hb9 ^+^/Hoxc9 ^+^ or *Tbx20 ^+^*/Isl ^+^ MNs—a MN profile characteristic of the rSC ( Figure 10D and Table 1). Finally, we assessed the MN subtype generated from stage 4 FB cells which generate ***Hoxb4 ^+^*/** *b8 ^−^/c9 ^−^* cHB progenitor cells, upon Wnt3A (150 ng/ml) and RA (10 nM) exposure. Addition of Shh-N (15 nM) induced *Tbx20 ^+^*/Isl ^+^, but only a few Hb9 ^+^/Isl ^+^ MNs and no Hb9 ^+^/Hoxc9 ^+^ MNs ( Figure 10C and Table 1)—a profile characteristic of cHB (r7 and r8) MNs ( Figure 1C and 1D).

**Table 1:**
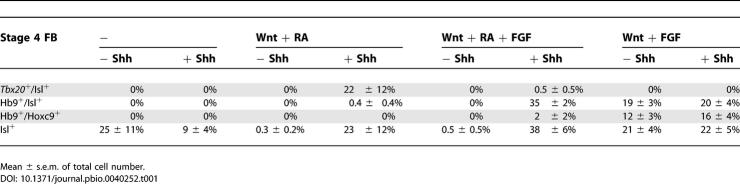
Quantification of Transcription Factor Expression in Neural Plate Explants

Collectively, these findings provide evidence that Wnt signals in combination with RA and/or FGF exposure, induce *Hox* profiles in neural cells that predict the later position-specific emergence of dMNs and vMNs characteristic of the hindbrain and spinal cord. These observations support the idea that early exposure to Wnt signals, together with later RA and FGF signals imposes *Hox* profiles that anticipate the patterned generation of dMN and vMN subclasses in the developing hindbrain and spinal cord.

## Discussion

This study has examined the link between extrinsic patterning signals, regionally restricted profiles of transcription factor expression in neural progenitor cells, and the specification of MN subtype along the rostrocaudal axis of the hindbrain and spinal cord. Our results support four main conclusions: (i) Wnt signaling is required to specify cells of spinal cord character, (ii) the initial specification of spinal cord progenitor cells appears to require prolonged, or higher level Wnt signaling than does the specification of cells of hindbrain character, (iii) early Wnt signaling provides a positional context for the later actions of RA and FGF signals in specifying the rostrocaudal regional identity of hindbrain and spinal cord cells, and (iv) the interplay of Wnt, retinoid, and FGF signals establish distinction in progenitor cell *Cdx* and *Hox* profiles that anticipate the rostrocaudal position of generation of dMNs and vMNs in the hindbrain and spinal cord. Below, we discuss the evidence that supports each of these conclusions.

### Interplay between Early Wnt and Later FGF and RA Signals in the Assignment of Caudal and Neural Fates

The generation of different subclasses of MNs along the rostrocaudal axis of the hindbrain and spinal cord depends on two crucial early steps of caudal neural development: first, the early specification of cells of hindbrain and spinal cord character, and second, the subsequent refinement of rostrocaudal regional character of hindbrain and spinal cord progenitor cells. Wnt signaling has been implicated in the generation of caudal neural cells [ 48, 60– 69], and results in chick have provided evidence that FGF and graded Wnt signaling in neural cells specify cells of caudal forebrain, midbrain, and rostral hindbrain character [ 32, 66]. The present study provides evidence that early Wnt signaling is also essential to impose caudal hindbrain and spinal cord character on neural progenitor cells. The results also support the view that the specification of spinal cord progenitor cells requires prolonged, or higher level Wnt signaling than is required for the specification of cells of hindbrain character, and they are consistent with previous findings that *Cdx* genes respond to Wnt signals and act upstream of 5′ *Hox* genes in neural progenitor cells [ 23, 70– 75]. Thus, taken together, our results support the idea that in the presence of FGF signals, graded Wnt signaling imposes midbrain, hindbrain, and spinal cord character on prospective caudal neural plate cells.

The signals and mechanisms that act in the subsequent step to impose rostrocaudal regional identity on hindbrain and spinal cord progenitor cells have been examined previously. From early somite stages, RA supplied by the paraxial mesoderm and newly formed somites, promotes the expression of *Hox* genes characteristic of the cHB and rostral levels of the spinal cord [ 6, 8, 29, 31, 67, 76, 77]. FGF signals derived from the regressing primitive streak promote the expression of progressively more caudal Hox-c proteins in a concentration-dependent manner [ 7, 8]. Thus, RA and FGF signals act in an opponent manner to impose rostrocaudal regional identity on hindbrain and spinal cord progenitor cells [ 6, 78]. Our findings extend these results by showing that early Wnt signaling establishes a positional context for the later actions of RA and FGF signals in specifying hindbrain and spinal cord cells of rostral and caudal regional identity.

**Figure 11:**
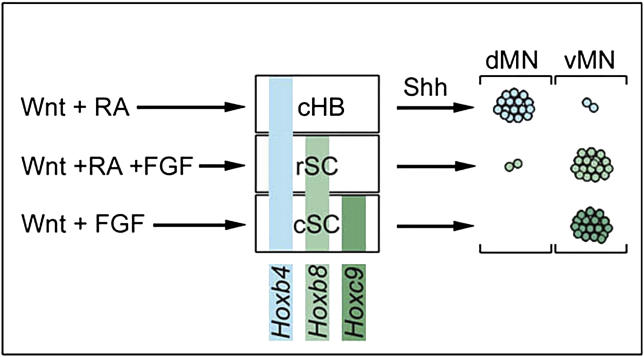
Combinatorial Wnt, RA, and FGF Signals Specify Progenitor Cell Identity That Prefigure MN Subtype in the Developing Hindbrain and Spinal Cord Combinatorial actions of Wnt, FGF, and RA signals specify neural progenitor cells expressing *Hox* gene profiles characteristic of the cHB, rSC, and cSC that generate patterns of differentiated MNs, with dMN or vMN exit points, characteristic of hindbrain and spinal cord, in response to Shh signaling.

Collectively, our results suggest a model of how hindbrain and spinal cord cells of early rostrocaudal regional identity are generated ( Figure 11). At gastrula stages, prospective caudal neural plate cells are exposed to Wnt signals derived from the emerging caudal paraxial mesoderm and from epiblast cells; and to FGF signals derived from the primitive streak [ 32, 46, 47, 79, 80]. In response to convergent Wnt and FGF signaling, prospective caudal neural plate cells are initially specified either as cells of rHB character or as ***Hoxb4 ^+^/b8 ^+^*/ *c9 ^+^*** cells characteristic of caudal/thoracic spinal cord. At early somite stages, caudal paraxial mesoderm and newly formed somites located adjacent to the prospective cHB and rSC, express high levels of *Raldh2,* providing a local source of RA [ 59, 81]. Our results suggest that RA specifies cells of r7/r8 cHB character by inducing *Hoxb4* expression in prospective rHB cells and cells of rSC identity by preventing the expression of *Hoxc9* in prospective cSC cells. At these stages, several *Fgfs* are expressed in the regressing Hensen's node and primitive streak adjacent to the developing spinal cord [ 8, 46, 79]. We find that FGF signals maintain the specification of cSC cells, and in the presence of RA, promote the generation of rSC cells. This model is strengthened by our data providing evidence that distinct combinations of Wnt, RA, and/or FGF signals can reconstitute rostral and caudal hindbrain and spinal cord character in naive prospective FB cells in a predictable manner.

Genetic analyses have provided evidence that inactivation of *Wnt* genes, expressed in the caudal regions of gastrula stage mouse and zebrafish embryos, perturbs the development of the caudal neural plate [ 60, 62, 63, 69, 82]. However, the formation of paraxial mesoderm, which serves as a local source of neural caudalizing signals [ 63, 83– 85], is also impaired in these mutant embryos [ 45, 69, 86]. Thus, these genetic studies left unresolved the issue whether the effect of perturbed Wnt signaling on caudal neural development reflects the impaired formation of paraxial mesoderm or reflects direct Wnt signaling in neural cells. Our in vitro studies establish direct effects of Wnt signals on neural tissue in the absence of other tissues and clarify the integrative mechanisms that control the early development of the hindbrain and spinal cord.

FGF and retinoid signals also regulate the temporal pattern of differentiation of caudal neural progenitor cells. FGF has been shown to keep cells in a stem zone–like state [ 14, 78, 87], whereas RA promotes the differentiation of neural cells [ 14, 78, 88]. Consistent with these roles of FGF and RA, exposure of naive neural explants, cultured with Wnt and FGF to low levels of RA (2 nM), does not change the rostrocaudal identity of neural progenitor cells but results in an increased number of differentiated MNs (unpublished data), whereas under these conditions, increased levels of FGFs greatly reduce MN differentiation (unpublished data). These findings fit well with the suggested opponent activities for FGF and RA in deciding the balance between neural cell proliferation and differentiation [ 87].

### *Hox* Gene Profiles of Hindbrain and Spinal Cord Progenitor Cells Predict the Pattern of dMNs and vMNs

The patterned expression of *Hox* genes in neural progenitor cells appears to be a major determinant of the identity of different MN populations [ 1, 7, 11, 16, 18]. Earlier studies have provided evidence that RA and FGF act in an opponent manner on caudal neural cells to establish the rostrocaudal pattern of distinct subclasses of differentiated MNs [ 7– 9]. dMNs and vMNs represent two major MN subclasses that are generated in distinct rostrocaudal patterns in the hindbrain and spinal cord [ 10, 89]. Our findings provide evidence that Wnt signaling in neural progenitor cells is required for the generation of both vMNs and dMNs. We also show that Wnt, RA, and/or FGF signals can induce cells with *Hox* gene profiles characteristic of cHB, rSC, and cSC progenitor cells that differentiate into corresponding dMNs and vMNs when exposed to Shh-N. These findings therefore reveal a tight link between Wnt, RA, and FGF signals, profiles of progenitor cell *Hox* expression, and the rostrocaudal pattern of dMN and vMN generation in the hindbrain and spinal cord.

Other recent studies have revealed a determinative role of *Hox* genes in MN subtype specification. In the hindbrain, Hoxb1 is expressed throughout r4, and has been shown to be required for the specification of facial branchiomotor neurons [ 17– 19]. Similarly, targeted expression of Hoxa3 in the rHB leads to the generation of ectopic somatic MNs [ 11]. In the spinal cord, the rostrocaudal profile of genes of the Hox6 to Hox9 paralog group have been shown to establish distinctions in MN columnar and pool subtype [ 7, 16]. It seems likely, therefore, that the initial profiles of *Hox* expression, shown here to depend on early Wnt signaling, are involved in establishing domains of dMN and vMN formation. Furthermore, *Hox* genes appear to represent a common regulatory target for the three classes of signaling factors—Wnts, FGFs, and RA [ 90]—that conspire to regulate the position of generation of dMNs and vMNs.

Thus, our results reveal that an early Wnt-based program is required to interact with a later RA- and FGF-mediated mechanism to generate a pattern of neural progenitor cells with *Cdx* and *Hox* profiles that prefigures the generation of two major subclasses of MNs in the developing hindbrain and spinal cord. Further studies will reveal how these three signals are integrated at the molecular level to regulate *Cdx* and *Hox* gene profiles, leading to the subsequent differentiation of dMN and vMN classes.

## Materials and Methods

### Embryos.

Fertilized white leghorn chicken eggs were obtained from Agrisera AB, Umeå, Sweden. Chick embryos were staged according to Hamburger and Hamilton (HH) [ 91].

### Isolation of tissue explants.

Prospective neural plate explants were isolated from HH stage 4, 5, 6, 7 (1-somite), and 8 (3–4-somites) chick embryos. For stage 5–8 embryos, Dispase I (Roche Diagnostics) was used to facilitate removal of the underlying mesoderm.

### Culture of tissue explants.

Explants were cultured in vitro as previously described [ 32]. To enable tracing the rostrocaudal orientation of the dissected explants, rostrocaudally asymmetrical explants were isolated and then placed in a defined orientation in collagen matrix, where their orientation was maintained during cultivation, fixation, and cryo-sectioning (see Figure 5A); or the caudal margin of the explants was labeled with DiI crystals (Molecular Probes) during the dissection process ( Figures 5D, 7B, 7C, and 7D). Recombinant human FGF4 (R & D Systems, Minneapolis, Minnesota, United States) was used at 30 and 60 ng/ml. The FGF receptor inhibitor SU5402 (Calbiochem, EMD Biosciences, San Diego, California, United States) was used at 3 and 5 μM. All- *trans* retinoic acid (RA) (Sigma-Aldrich, St. Louis, Missouri, United States) was used at 10–40 nM. Purified recombinant mouse Wnt3A (R & D Systems) was used at 150 ng/ml. Soluble Wnt3A and control-conditioned media [ 92] were obtained as described [ 32] and used at 50–100 μl/ml, which for Wnt3A conditioned medium, mimicked the activity 75–150 ng/ml of Wnt3A protein (R & D Systems). Soluble mouse Frizzled 8 (mFrz8CRD-IgG) [ 50] and control-conditioned medium were generated as described [ 32] and used at 300–500 μl/ml of culture medium. Explants cultured with control-conditioned medium behaved like explants cultured alone. In MN differentiation studies, Shh-N (R & D Systems) was used at 15 nM, and cultivated explants were washed to remove Wnt, RA, and/or FGF4 from the medium before addition of Shh-N.

### In situ hybridization, immunohistochemistry, and quantification of cells.

Chick *Hoxb4, Hoxb8, Hoxc9, CdxC, CdxB, Tbx20,* and *Mox1* (see Figure S3, [ 55]) and *Brachyur*y ( *Bra)* (see Figure S3, [ 56]) expression was detected using in situ RNA hybridization on consecutive cryo-sections using digoxigenin-labeled probes and was carried out essentially as described [ 93], before being labeled with DAPI for quantification. Hoxc9 protein and all further markers were detected using fluorescent immunohistochemistry. Neural tissue was detected with rabbit anti-Sox1 antiserum, kindly provided by S. Wilson. Rabbit anti-Otx2 was kindly provided by G. Corte. Rabbit anti-Krox20 was obtained from Babco. Rabbit anti-Isl1/2, mouse anti-Hb9, and rabbit anti-Hoxc9 were used as described [ 8, 94]. Co-localization of *Tbx20*/Isl was determined by double labeling with RNA probe and antibody, and co-localization of Hb9/Isl and Hoxc9/Hb9 was determined by double labeling with antibodies. Images of consecutive sections were collected using a Nikon (Tokyo, Japan) E800 light/epi-flourescent microscope. Images presented are representative of the number of explants indicated, for each experiment, in the figure legends. For each experiment, all explants were sectioned, and cells from consecutive sections were counted from 3–5 representative explants using nuclear Dapi staining. Each explant usually generated ˜10–18 8-μm sections, and the cell counts in Figures 3, 7, and 9 indicate the mean percentage of total cell number ± s.e.m. per section in *Hox*-gene ^+^ domains that were compared by overlay of consecutive sections. The number of Isl1/2 ^+^ cells, *Tbx20 ^+^*/ Isl1/2 ^+^ cells, Hb9 ^+^/ Isl1/2 ^+^ cells, and Hb9 ^+^/ Hoxc9 ^+^ cells in each experiment was acquired from two to four explants (two sections from each explant) as described above ( Figures 4, 6, and 10).

## Supporting Information

Figure S1 Wnt3A or FGF4 Alone Does Not Induce More Caudal Character in Prospective rHB Cells (A) Schematic drawing of a stage 4 embryo. Dotted line indicates the presumptive neural plate. Boxed region indicates caudal (C) neural plate explants used for in vitro studies.
(B–D) Stage 4 C explants cultured alone (B), in the presence of FGF4 (120 ng/ml) ( *n* = 18 explants) (C), or Wnt3A (150 ng/ml) ( *n* = 9 explants) (D) for 44 h generated a domain of Krox20 ^+^ cells (30%–60% of total cell number) and a domain of *Hoxb4* ^+^ */Hoxb8* ^+^ */Hoxc9* ^+^ cells (40%–70% of the total cell number). Each row represents consecutive sections from a single explant. Scale bar represents 100 μm.
(3.2 MB TIF)

Figure S2 Wnt, FGF or RA Signals Alone Do Not Induce More Caudal Character in Prospective FB Cells (A) Schematic drawing of a stage 4 embryo. Dotted line indicates the presumptive neural plate. Red box indicates prospective FB explants that were used for in vitro studies. (B–F) Sox1 was used as a general neural marker. Each row represents consecutive sections from a single explant.
(B–F) Stage 4 FB explants cultured alone ( *n* = 24 explants) (B), or in the presence of Wnt3A (75 ng/ml) ( *n* = 12 explants) (C), FGF4 (60 ng/ml) ( *n* = 9 explants) (D), RA (10 nM) ( *n* = 17 explants) (E), or FGF (60 ng/ml) and RA (10 nM) in combination ( *n* = 12 explants) (F) for 44 h generated Sox1 ^+^/ Otx2 ^+^ cells (75%–98% of total cell number), but no caudal neural cells. Scale bar represents 100 μm.
(3.1 MB TIF)

Figure S3 Wnt3A, FGF4, and RA in Combination Do Not Block Neural, or Induce Mesodermal Character, in Prospective FB or Caudal Neural Cells (A) Schematic drawing of a stage 4 chick embryo. Dotted line indicates the presumptive neural plate. Red box indicates FB explants and black box indicates caudal neural plate tissue explants (4C) that were used for in vitro studies.
(B–D) All explants were cultured for 24 h (˜ corresponding to a stage 10 embryo)
(B) Stage 4 FB explants exposed to Wnt3A (150 ng/ml), FGF4 (20ng/ml), and RA (10 nM) generated Sox1 ^+^ and Sox2 ^+^ cells, but no *Mox1-* or *Bra-*expressing cells.
(C) Stage 4 FB explants exposed to Wnt3A (150 ng/ml) and FGF4 (60 ng/ml) generated Sox1 ^+^ and Sox2 ^+^ cells and few *Bra* ^+^ cells, but no *Mox1-*expressing cells.
(D) Stage 4C explants grown in the presence of Wnt3A (˜75 ng/ml) and FGF4 (60 ng/ml), in combination, generated Sox1 ^+^ and Sox2 ^+^ cells and few *Bra* ^+^ cells, but no *Mox1-*expressing cells.
(E–G) Transversal sections of a stage 10 embryo.
(E) Schematic drawing of a stage 10 embryo. The lines indicate the level of the transverse sections shown in the corresponding panels (F and G).
(F) Sox1 and Sox2 are expressed in the neural tube. *Mox1* is expressed in the adjacent somites (s), whereas *Bra* is expressed in the notochord (NC).
(G) Sox1 and Sox2 are expressed in the neural plate (NP). *Mox1* is expressed in the presomitic mesoderm (PSM) at the neural plate level and *Brachyury* is expressed in the neural plate and in the mesendoderm.
(3.1 MB TIF)

## Abbreviations

C: caudal
cHB: caudal hindbrain
CN: caudal to the node
cSC: caudal spinal cord
dMN: dorsal exiting motor neuron
FB: forebrain
FGF: fibroblast growth factor
HH: Hamburger and Hamilton
MN: motor neuron
NL: node level
r: rhombomere
RA: retinoic acid
RALDH2: retinaldehyde dehydrogenase 2
rHB: rostral hindbrain
RN: rostral to the node
rSC: rostral spinal cord
Shh: sonic hedgehog
Shh-N: N-terminal fragment of the sonic hedgehog protein
vMN: ventral exiting motor neuron
